# *Methylobacterium extorquens* PA1 utilizes multiple strategies to maintain formaldehyde homeostasis during methylotrophic growth

**DOI:** 10.1371/journal.pgen.1011736

**Published:** 2025-06-09

**Authors:** Zachary T. Hying, Anya M. Rushmer, Chin Yi Loh, Eric L. Bruger, Jannell V. Bazurto

**Affiliations:** 1 Department of Plant and Microbial Biology, University of Minnesota Twin Cities, St. Paul, Minnesota, United States of America; 2 Biotechnology Institute, University of Minnesota Twin Cities, St. Paul, Minnesota, United States of America; University of Wisconsin-Madison, UNITED STATES OF AMERICA

## Abstract

Metabolic homeostasis is a central organizing principle of physiology whereby dynamic processes work to maintain a balanced internal state. Highly reactive essential metabolites are ideally maintained at equilibrium to prevent cellular damage. In the facultative methylotrophic bacterium *Methylobacterium extorquens* PA1, the utilization of one-carbon growth substrates, including methanol, generates formaldehyde as an obligate intermediate. Formaldehyde is highly chemically reactive and capable of damaging various biomolecules, making formaldehyde homeostasis critical during methylotrophic growth. However, homeostatic mechanisms that govern formaldehyde balance, which is readily perturbed upon transitioning to methylotrophic growth substrates, have remained elusive. Here we describe how a formaldehyde-sensing protein EfgA, a formaldehyde-responsive MarR-like regulator TtmR, and lanthanide-mediated methylotrophy together impact formaldehyde balance and one-carbon metabolism more broadly when cells are transitioning to growth on formaldehyde-generating one-carbon sources. We found that cells lacking *efgA* or *ttmR* are unable to maintain formaldehyde balance during various carbon source transitions resulting in elevated extracellular formaldehyde concentrations and an extended lag phase. In strains lacking *efgA*, we showed that inflated intracellular formaldehyde pools were accompanied by decreased cell viability, while the loss of *ttmR* resulted in the loss of one-carbon metabolites to the extracellular space. Additionally, we found less severe formaldehyde imbalances in the presence of lanthanides, even in the absence of *efgA* and *ttmR.* This was partly due to the activation of *exaF*, a lanthanide-dependent alcohol dehydrogenase that served as an alternative formaldehyde-detoxifying system that lessened the necessity of *ttmR* for maintaining formaldehyde homeostasis. Overall, our data demonstrated that *efgA* has a primary role in formaldehyde homeostasis in modulating intracellular formaldehyde pools, while *ttmR* is secondary, preventing carbon loss to the extracellular space. These results led us to develop a model of formaldehyde homeostasis involving formaldehyde sensing, growth arrest, compartmentalization, and auxiliary detoxification systems. This work deepens our understanding of how physiological factors impact biological formaldehyde homeostasis during transient metabolic imbalances of this universal cellular toxin.

## Author summary

In our study, we explore how the bacterium *Methylobacterium extorquens* PA1 manages formaldehyde, a natural product of metabolism that is often viewed as a harmful toxin, but which also plays essential roles in biological processes. We focus on two key proteins, EfgA and TtmR, which help the bacterium transition to using methanol as a carbon (i.e., food) source. Our findings reveal that these proteins work independently to control formaldehyde levels, ensuring the bacterium can grow and thrive without being overwhelmed by this potentially harmful substance. Additionally, we show that metals known as lanthanides, along with a particular enzyme called ExaF, help the bacterium handle formaldehyde more efficiently. These insights not only deepen our understanding of how bacteria manage formaldehyde but also highlight potential applications in biotechnology. Moreover, understanding these natural processes may have implications for human health, as formaldehyde is also present in human cells and linked to various diseases. By harnessing these natural processes, scientists could improve industrial processes that rely on methanol, reduce environmental impact, or even use bacteria to produce valuable chemicals. Our work underscores the importance of re-evaluating chemicals traditionally seen as harmful, recognizing their potential benefits in biological systems.

## Introduction

Metabolic homeostasis is the tendency for metabolites to be maintained at relatively constant concentrations that support the growth and viability of the cell and is a central organizing principle of physiology [1]. Changes in environmental or regulatory conditions can perturb metabolic homeostasis and trigger cellular responses that return the cell to homeostatic conditions. Deficiencies in metabolic homeostasis are linked to numerous defects and cell death [2,3]. Conversely, robust systems of metabolic homeostasis can help maintain intracellular conditions despite external fluctuations in the environment [4]. Bolstering homeostatic systems has important biotechnological applications such as increased metabolic stability, which can improve stress tolerance, optimize resource utilization, and ultimately increase genetic stability [5,6].

Reactive metabolites, although necessary for driving metabolism forward, also often have the potential to damage important biomolecules. Thus, maintaining reactive metabolites at homeostatic concentrations is a major cellular priority. One such reactive metabolite, formaldehyde (FA), is a naturally occurring and universally toxic metabolite [7]. FA can damage a myriad of biological molecules, including DNA and proteins, leading to cellular stress and even death. Notably, elevated FA has also been implicated in numerous disease states in higher organisms including diabetes, neurological disorders, and cancer [8–12]. As such, all domains of life encode FA detoxification systems [13–15], which are often activated via FA-sensing transcription factors, including FrmR in *Escherichia coli* [16] and HxlR in *Bacillus subtilis* [17].

However, the role of FA extends beyond that of just a toxin: biologically produced FA is essential in some scenarios, such as in methylotrophic metabolism in microbes, and numerous beneficial roles for FA have been recently reported in higher organisms. In mammals, purine biosynthesis is supported by FA-derived formate, where FA is produced as a byproduct of one-carbon (C_1_) metabolism that is then detoxified [15]. Because formate is required for cells to produce nucleotides, amino acids, and methylation reactions, and the oxidation of FA to formate is a prevalent detoxification mechanism, FA is potentially required in numerous beneficial biological processes in a wide breadth of organisms. Additional studies have demonstrated that finely tuned FA levels are required for memory formation, where both excess and lack of FA cause detrimental cognitive defects. This overall suggests that FA levels are both carefully sensed and controlled by cells [18]. As our understanding of the role FA has in biology expands, it is crucial to develop model systems to gain a deeper insight into how cells sense and maintain sub-lethal homeostatic FA concentrations and allow beneficial processes to occur.

Methylotrophic bacteria are a promising model to study FA homeostasis because of their formaldehyde-centric metabolism. Methylotrophs such as *Methylobacterium extorquens* can utilize reduced C_1_ carbon compounds such as methanol (MeOH) as a sole source of carbon and energy [19,20]. *M. extorquens* first oxidizes MeOH to FA using either a calcium- (MxaFI) or lanthanide-dependent (XoxF) MeOH dehydrogenase in the periplasm, making FA an obligate intermediate of this metabolism [20,21]. FA then enters the cytoplasm where concentrations are maintained in the low millimolar range [22,23] by formaldehyde activating enzyme (FAE), which condenses FA with the C_1_ carrier dephosphotetrahydromethanopterin (H_4_MPT). A series of H_4_MPT-linked reactions oxidizes FA and ultimately produces formate. This is considered a critical branchpoint of the methylotrophic pathway, in which formate can either be assimilated into biomass or completely oxidized as an electron source [24–26]. For this metabolism to continue successfully, the concentration of FA must be sufficiently high to support the forward reaction of FAE that allows carbon utilization but also sufficiently low to prevent cellular damage and lethality. Intracellular concentrations (combined periplasm and cytoplasm) of FA have been measured in the most extensively studied methylotroph *M. extorquens* AM1 at ~500µM during growth on MeOH [23].

While much is known about the enzymatic steps and biochemistry involved in *M. extorquens* FA-centric metabolism, little is understood about how *M. extorquens* responds to and maintains intracellular concentrations of FA. Recent work in the strain *M. extorquens* PA1 has identified two genes, *efgA* and *ttmR*, which are involved in the cellular response to FA produced during methylotrophic metabolism [22,27,28]. Loss-of-function mutations in either gene leads to increased FA tolerance at the expense of an optimal transition to methylotrophy. The defective transitions in the mutant strains are accompanied by elevated release of FA into the medium, suggesting metabolic imbalance.

Overall, *M. extorquens* appears to have several layers of physiology in place to maintain FA at homeostatic levels. The *efgA* gene encodes a conserved FA sensor that halts translation in response to elevated FA levels leading to growth arrest [22,28], while the deletion of *efgA* forgoes translational stalling and enables *M. extorquens* to utilize FA as a sole source of carbon and energy. Additionally, *efgA* can be complemented by homologs from other species, and its expression increases FA tolerance in non-methylotrophs [22]. *ttmR* encodes a MarR-family transcription factor that impacts the regulation of numerous genes, including several other regulatory proteins, stress responses, and signaling pathways. *ttmR* modulates FA tolerance independently of *efgA* and deletion of both genes increases FA tolerance compared to either single mutant [27]. The specific mechanism of FA tolerance by *ttmR* remains undescribed. Research in closely related *M. extorquens* AM1 has also revealed that the *exaF* gene, which encodes a lanthanide-dependent periplasmic alcohol dehydrogenase, can improve growth under heightened endogenous FA conditions [23]. Despite the obvious impact of these factors on the cellular response to FA, there has yet to be a thorough analysis of their combined ability and individual contributions to support FA homeostasis.

Our systematic approach reveals that the interplay between genetic and environmental factors, such as EfgA, TtmR, and lanthanides, is vital for maintaining formaldehyde homeostasis during metabolic transitions. Our results show that cells lacking *efgA* or *ttmR* are unable to maintain FA homeostasis during transitions to carbon sources where FA naïve cells are suddenly faced with elevated FA, including but not limited to the transition to methylotrophy. Additionally, lanthanide-based methylotrophy adds a layer of cellular control that reduces the transient FA imbalance by activating ancillary FA detoxification mechanisms, but does not alleviate the metabolic imbalance, rather shifting the imbalance downstream to formate. By defining the impacts of these multilayered strategies, we provide a model of FA homeostasis in the model methylotroph *Methylobacterium extorquens* PA1 that describes the physiological management of cellular FA levels, and not only enhances our understanding of methylotrophic metabolism but also offers insights into formaldehyde regulation with potential implications for biotechnological and health-related applications. Further, this work highlights the adaptive capabilities of methylotrophs in managing reactive intermediates. As formaldehyde continues to emerge as a key player in both microbial and higher organism biology, our findings facilitate harnessing these pathways for practical innovations.

## Results

### EfgA and TtmR independently mitigate FA imbalance during the transition to methylotrophy

During the transition to methylotrophy, strains lacking either *efgA* or *ttmR* have comparable extended lag phase defects and suffer FA imbalance, as determined by increased FA release into the supernatant [27]. To determine whether EfgA and TtmR act independently or are redundant parts of a common underlying process, wild-type (WT), each single mutant (*ΔefgA* and *ΔttmR)*, and the double mutant (*ΔefgA ΔttmR*) were transitioned to methylotrophy via a carbon source switch experiment (S1 Fig). Here, stationary phase cells grown on succinate were inoculated into fresh medium supplemented with MeOH_._ We assayed growth and extracellular FA at the end of lag phase for each strain. Hereon, cells experiencing a new transition to methylotrophy are described as ‘FA naïve’ and cells already acclimated to growth on MeOH as a carbon source are described as ‘FA acclimated’.

**Fig 1:**
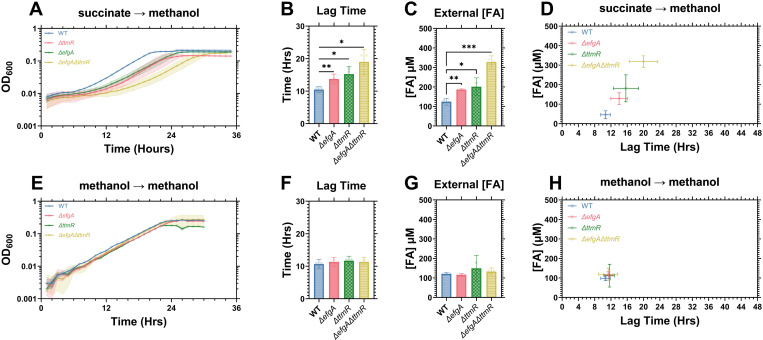
*efgA* and *ttmR* affect the transition to methylotrophy independently. The carbon source of FA naïve cells was transitioned from succinate to MeOH [A-D] whereas the carbon source of FA acclimated cells were only grown on MeOH (no transition) [E-H]. Growth [A,E], of *M. extorquens* PA1 strains (WT, blue; *ΔefgA*, red; *ΔttmR*, green; *ΔefgA ΔttmR*, yellow) was assayed, and their lag times [B,F], and extracellular FA concentrations at the end of lag phase [C,G] was measured. The correlation between extracellular formaldehyde concentration and lag phase duration [D,H]. Error bars represent the 95% confidence interval of three biological replicates. Statistical significance was determined using a Brown-Forsythe and Welch’s ANOVA. * = p < 0.05, ** = p < 0.01. *** = p < 0.001.

Consistent with previous results, the FA naïve *ΔefgA* and *ΔttmR* single mutants had comparable lag phase defects and elevated FA concentrations in MeOH media relative to WT (**Fig 1**A-D). In the *ΔefgA ΔttmR* double mutant, we observed an additive exacerbation of both the lag phase defect and increased extracellular FA levels (**Fig 1**A-D), demonstrating that both mutations independently contributed to the phenotypes. In FA-acclimated cells we observed no significant change in either lag phase duration or extracellular FA concentration (**Fig 1**E-H). Together these data illustrate that *efgA* and *ttmR* independently contribute to the optimal transition to methylotrophy and minimize the FA imbalance that occurs. Further, these data reveal a positive correlation between the external FA concentrations and the severity of lag phase defects (**Fig 1D**) that exists only in the transition to methylotrophy and not for FA-acclimated cells (**Fig 1H**).

### The lag phase defect is specific to carbon transitions that generate FA as an essential intermediate

**Fig 2:**
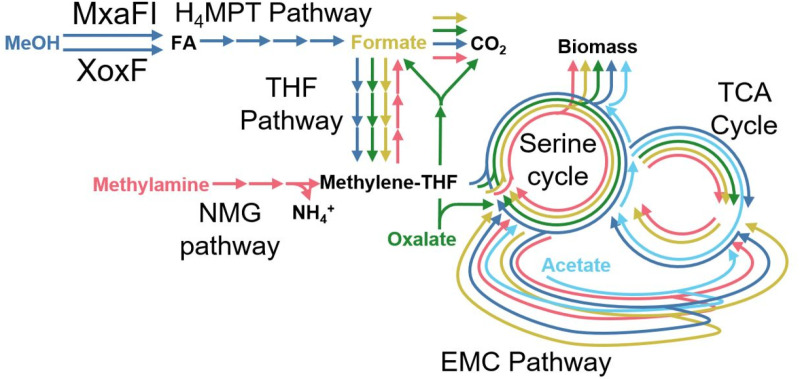
Diagram of metabolic pathways for C_1_ and C_2_ growth substrates in *M. extorquens* PA1. Diagram of *M. extorquens* PA1 central metabolism depicting pathways required the utilization of MeOH (dark blue arrows), methylamine (pink arrows), formate (yellow arrows), oxalate (green arrows), or acetate (light blue arrows). Abbreviations: MxaFI:calcium-dependent methanol dehydrogenase, XoxF:lanthanide-dependent methanol dehydrogenase, H_4_MPT:dephosphotetrahydromethanopterin, THF:tetrahydrofolate, NMG:N-methylglutamate, EMC:ethylmalonyl-CoA, TCA:tri-carboxylic acid. MeOH:methanol, FA:formaldehyde.

Given that elevated FA positively correlates with the severity of lag phase defects during the transition to methylotrophy in *efgA* and *ttmR* mutants, we hypothesized that the lag defects would be unique to carbon source transitions that generated FA as an obligate intermediate. Additionally, since the transition from succinate to MeOH also involves the activation of four metabolic pathways: the H_4_MPT-dependent formaldehyde dissimilation pathway, the tetrahydrofolate (THF) pathway, the serine cycle and the ethylmalonyl-CoA (EMC) pathway (**Fig 2**, blue arrows), it is a formal possibility that the mutations confer one or more pathway defects that contribute to the overall prolonging of the lag phase. To disentangle the production of FA from the potential contributions of each newly activated pathway to the lag defect, we conducted a series of carbon switch experiments with acetate, oxalate, formate, and methylamine [29–31].

We first assessed the transition from succinate to the C_1_ substrates formate or methylamine, neither of which leads to substantial FA production in *M. extorquens* PA1 (**Fig 2**, yellow and pink arrows, S2A-D Fig). Under these conditions, no lag phase defects were observed in any of the mutant strains (S2A-D Fig), demonstrating that *efgA* and *ttmR* have no effect on transitions to methylotrophy when the carbon substrate does not produce FA as an obligate intermediate. This reinforced the connection between the lag phase defect and excess FA produced during lag phase.

**Fig 3:**
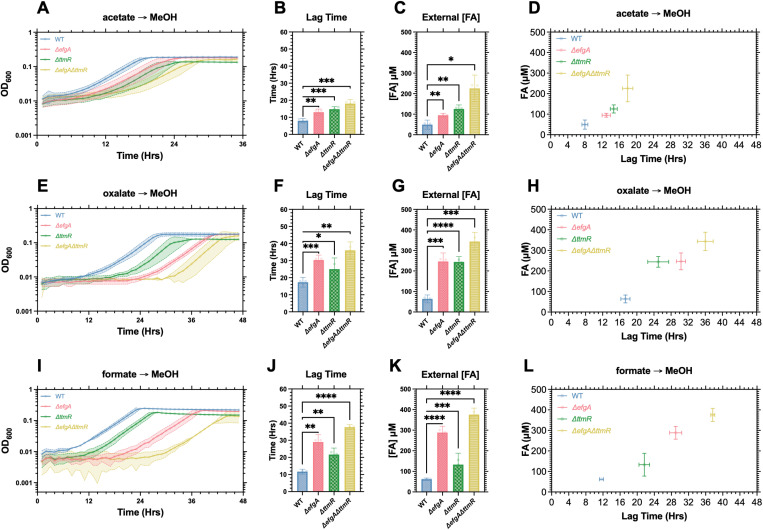
The lag phase defect is specific to transitions that require the production of FA, and correlates with FA concentration. A,E,I) Growth of *M. extorquens* PA1 strains (WT, blue; *ΔefgA*, red; *ΔttmR*, green; *ΔefgA ΔttmR*, yellow) during carbon source transition from acetate [A], oxalate [E] or formate [I] to MeOH. B,F,J) Lag times of each strain. C,G,K) Extracellular FA concentration at the end of lag phase for each strain. D,H,L) Correlation between extracellular FA concentration and duration of lag phase. Error shading and bars represent the 95% confidence interval of three independent biological replicates. Statistical significance was determined using a Brown-Forsythe and Welch’s ANOVA. * = p < 0.05, ** = p < 0.01, *** = p < 0.001.

Next, we assessed the potential contributions of activating the EMC and THF pathways and the serine cycle by transitioning succinate-grown cells to acetate or oxalate, neither of which are methylotrophic (i.e., FA-generating) growth substrates (**Fig 2**, light blue and green arrows). Acetate utilization in *M. extorquens* requires the EMC pathway whereas oxalate utilization requires the THF pathway and serine cycle [29,30]. Again, we observed no lag phase defects in any of the mutant strains (S2E-H Fig), suggesting that neither utilization of the serine cycle nor the EMC pathway cause the lag phase defects observed in the succinate to MeOH transition. Conversely, lag phase defects occurred when switching cells from growth on acetate, oxalate, or formate to MeOH (**Fig 3**), demonstrating that the defect is not singularly specific to the succinate to MeOH transition. In these defect-manifesting transitions, like the succinate to MeOH transition, we observed increased external FA concentrations that positively correlated with the severity of the lag phase defects (**Fig 3**). Intriguingly, the FA accumulation and correlated lag phase defects were most severe when switching cells from formate to MeOH (**Fig 3**I-L), a C_1_-to-C_1_ transition that is only differentiated by the activation of the H_4_MPT pathway. Taken together, these results rule out suboptimal flux through the serine cycle and EMC pathway as possible root sources of FA accumulation and/or lag phase defects and demonstrate a broader role for *efgA* and *ttmR* beyond the transition to methylotrophy.

### Loss of EfgA disrupts FA homeostasis with broad consequences for C_1_ metabolism

After observing exacerbated FA imbalances when *efgA* or *ttmR* are deleted, we sought to investigate their impacts on C_1_ metabolism more broadly. We conducted a formate to MeOH switch experiment, as it represented a simplistic C_1_ to C_1_ transition, and measured key metabolites in MeOH utilization (**Fig 2**). Specifically, we measured the extracellular concentrations of MeOH (carbon source input), FA (toxic intermediate of the H_4_MPT pathway) and formate (branchpoint metabolite between dissimilatory and assimilatory pathways), as well as the intracellular concentrations of FA and formate over time. To accurately measure the intracellular concentrations of metabolites, high density cultures were achieved via inoculation at an initial OD_600_ of 0.2 rather than ~0.01 (S1 Fig).

**Fig 4:**
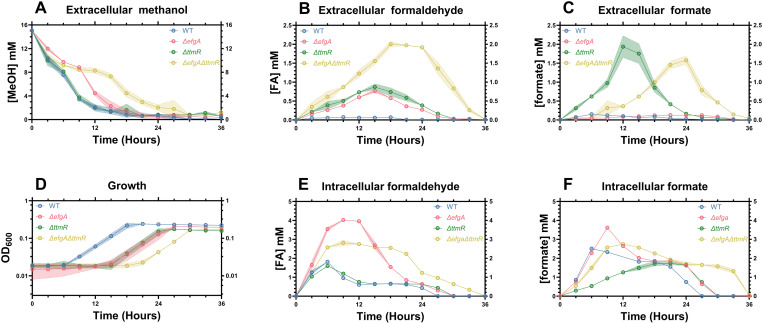
Concentrations of C_1_ metabolites are imbalanced in strains that cannot maintain FA homeostasis during the transition from formate to MeOH. Concentration of extracellular MeOH (A), extracellular formaldehyde (B), extracellular formate (C), optical density (D), intracellular formaldehyde (E), intracellular formate (F) of *M. extorquens* PA1 (WT [Blue], *ΔefgA* [Red], *ΔttmR* [Green], *ΔefgA ΔttmR* [Yellow]) cultures. Error shading represents the 95% confidence interval of the metabolite concentration of three independent biological replicates measured in technical triplicate. Peak metabolite values and associated statistical analysis can be found in S4 Table.

In the *ΔefgA* mutant, nearly all metabolites were altered compared to WT (Figs 4A-C, 4E, 4F **and** S3A-D), reflecting an overall defect in MeOH usage. MeOH consumption by the *ΔefgA* mutant was like WT for the first 6 h until it leveled off (~10 mM) for approximately 3 h, after which consumption steadily resumed until MeOH was undetectable (**Figs 4A**, S3C). The external FA concentration of the *ΔefgA* mutant increased steadily before peaking at 0.8 mM after 15 h compared to a peak of 0.1 mM after 3 h in WT. (**Figs 4B**, S3C). The internal FA concentration was also increased in *ΔefgA* relative to WT, peaking at approximately 4 mM after 9 h and then remaining constant until after 12 h (**Figs 4E**, S3D). This peak was both significantly higher and later than the peak of the internal FA concentration in WT, which reached about 1.8 mM after 6 h, before the cells reached a steady state during exponential growth (~0.6 mM, **Figs 4E**, S3D). External formate concentrations were in sub-millimolar ranges, similar to WT but steadily increased, reaching a maximum of approximately 100 µM between 20 and 27 h (**Figs 4C**, S3C). Internal formate reached a similar steady state concentration to WT of 1.8 mM between 15 and 24 h during exponential growth, but the initial imbalance was exacerbated in the *ΔefgA* mutant (maxima: *ΔefgA* = 3.5 mM, WT = 2.5 mM, **Figs 4F**, S3D).

These results suggest that the *ΔefgA* mutant cannot maintain internal FA homeostasis and releases excess FA due to unrestricted FA production and/or constrained consumption, leading to cellular overflow. To confirm that the loss of *efgA* disrupts FA homeostasis generally, intracellular FA was measured for all strains in each of the aforementioned defective carbon transitions to methylotrophy (S4 Fig). Indeed, intracellular FA pools were elevated in each defective transition, suggesting that the FA imbalance experienced by the Δ*efgA* mutant arose from the intracellular environment and lays the potential basis for the mutant strain’s growth defect in transitioning to methylotrophy.

### Loss of TtmR leads to loss of C_1_ metabolites to the environment

Despite some overlapping phenotypes, the transition from formate to MeOH revealed significant differences between the physiological impact of losing *ttmR*, compared to *efgA*. In the *ΔttmR* mutant, the consumption of MeOH was identical to WT (**Figs 4A**, S3A, S3E). Extracellular FA was elevated relative to WT, increasing steadily to a maximum near the entry into exponential growth phase (0.7 mM compared to 0.1 mM in WT) and then decreased steadily until it was undetectable at the entry into stationary phase (**Figs 4B**, S3E). Surprisingly, internal FA was comparable to WT (**Figs 4E**, S3F). Both extracellular and intracellular formate concentrations were notably different from WT. Extracellular formate increased dramatically for 12 h, peaking at 1.8 mM, more than an order of magnitude greater than the maximum of WT (0.12 mM), before steadily decreasing and becoming undetectable at the entry to stationary phase (**Figs 4C**, S3F). Intracellularly, unlike WT where the formate concentration peaked at 6 h, *ΔttmR* does not experience the initial imbalance but rather formate slowly increases to a maximum of ~1.5 mM between 15 and 18 h, where it remains constant until the cells reach stationary phase (**Figs 4F**, S3F). These results suggest that *ΔttmR* cells maintain internal FA homeostasis but release both FA and formate to the extracellular space, likely causing a delay in cells reaching a metabolic steady state that can support exponential growth.

Previous work with the *ΔttmR* mutant identified altered expression of genes encoding enzymes required for the synthesis of O-antigen and Lipid A sugar moieties and corresponding transglycosylases predicted to be involved in the assembly of lipopolysaccharide, a key component of the outer membrane [27]. To understand the physiological basis of imbalanced internal and external C1 metabolites, we performed real-time quantitative PCR analysis on representative genes from each of these operons *(Mext_3691, Mext_4619),* along with a series of genes required for methanol utilization, including genes encoding subunits for each of the four formate dehydrogenases. We observed significant down-regulation of *Mext_3691* and up-regulation of *Mext_4619* in strains lacking *ttmR* compared to WT (S5 Fig). Additionally, we observed significant up-regulation of the periplasmic formate dehydrogenase (*fdh3DABC*, *Mext_0388–0391*) in strains lacking *ttmR* but no changes in expression of the other formate dehydrogenases (*fdh1BA, Mext_4581–4582; fdh2CBAD, Mext_4404–4407; fdh4AB, Mext_2104–2105*). Among other metabolic genes surveyed, each representing a different portion of methanol utilization pathway, all showed a minor trend of decreased expression, though no changes were statistically significant (S5 Fig). These results suggest that FA-related defects observed in the *ttmR* mutant may result from compounding effects of physiological changes to the outer membrane in combination with increased formate oxidation.

### EfgA and TtmR have complementary roles in maintaining internal FA homeostasis and preventing loss of obligate intermediates of C_1_ metabolism

In the *ΔefgA ΔttmR* mutant, we observed a complex mixture of phenotypes. Like the *ΔefgA* mutant, the double mutant experienced a pause in MeOH consumption (**Figs 4A**, S3G); however, the ~ 9 h pause was significantly exacerbated compared to the ~ 3 h pause seen in the *ΔefgA* single mutant (**Figs 4A**, S3G). External FA was increased compared to WT or either single mutant, peaking from 21-24 h at ~2.0 mM (**Figs 4B**, S3G). Internal FA in the *ΔefgA ΔttmR* mutant was intermediate relative to the *ΔefgA* mutant and WT/*ΔttmR* strains but never reached a steady state concentration (**Figs 4E**, S3H). Thus, it appears that the double mutant cannot maintain internal FA homeostasis, like the *ΔefgA* mutant, nor can in prevent the loss of FA to the extracellular space, like the *ΔttmR* mutant. Finally, the intracellular and extracellular formate concentrations were both increased relative to WT, and further, the external formate peaked ~12 h later than in the *ΔttmR* single mutant (**Figs 4C**, **4F**, S3G, S3H).

The double mutant largely possesses phenotypes that are definitively additive compared to either of the single mutants, but also demonstrates the capacity for compounding phenotypes, as it suffers an extensive pause in MeOH consumption not seen in either single mutant. Taken as a whole, these results demonstrate the distinct importance of both *efgA* and *ttmR* in the maintenance of FA homeostasis and the effect their loss has, disrupting metabolic homeostasis and growth more broadly.

### Apparent lag defect is due to decreased viability in cells lacking *efgA*

**Fig 5:**
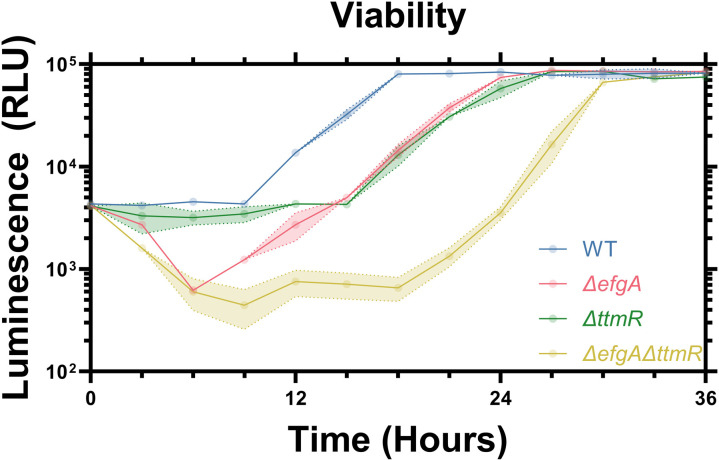
Cells lacking *efgA* lose viability before recovering upon the transition to MeOH utilization. Viability of *M. extorquens* strains (WT, blue; Δ*efgA*, red; Δ*ttmR*, green; *ΔefgA ΔttmR*, yellow) during transition from formate to MeOH. Error shading represents the 95% confidence interval of the metabolite concentration of three independent biological replicates measured in technical triplicate.

In addition to measuring key C_1_ metabolites during the formate to MeOH transition, we investigated how the changes in cell viability during carbon switches might contribute to the apparent extended lag defects in mutant strains (**Fig 5**). Here, we observed distinct strain-level differences, with WT and *ttmR* strains experiencing static cell counts during this period, while strains lacking *ΔefgA* demonstrated a notable and comparable drop in viability. Thus, we can attribute some portion of the apparently extended lag phase in strains lacking *efgA* to a loss of cell viability. The observed decreases in viability corresponded with the respective pauses in MeOH consumption in each strain, as well as with the periods of most severe intracellular FA imbalance (**Figs 4E**, **5 and** S3D). Together, these results suggest that cells lacking *efgA* succumb to FA toxicity at a heightened rate, leading to a drop in viability, while *ΔttmR* cells experience a true extension of lag phase.

### Detoxification of intracellular FA during exogenous FA stress occurs more quickly in cells lacking *efgA*

**Fig 6:**
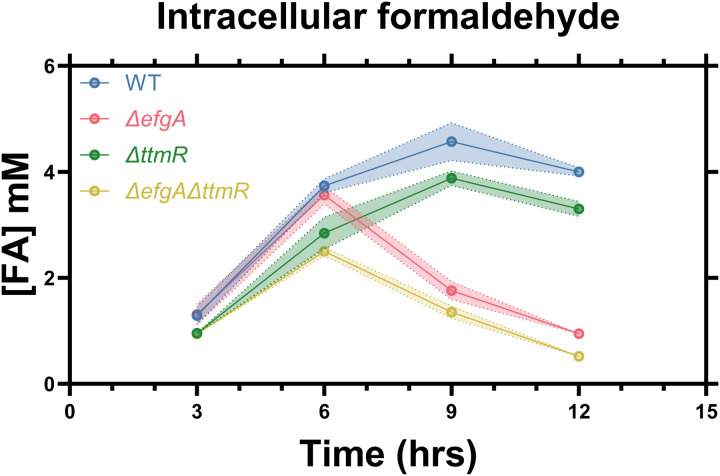
Cells lacking *efgA* detoxify intracellular FA during exogenous FA stress. Intracellular FA concentrations of *M. extorquens* strains (WT, blue; Δ*efgA*, red; Δ*ttmR*, green; *ΔefgA ΔttmR*, yellow) when inoculated into MP media containing 3.5mM succinate and 5mM FA as an exogenous stressor. Error shading represents the 95% confidence interval of the metabolite concentration of three independent biological replicates measured in technical triplicate.

Given that strains lacking *efgA* thrive under exogenous FA levels up to 8 mM [22], it seems counterintuitive that experiencing elevated internal FA concentrations resulted in loss of viability during the formate to MeOH transition. To address this apparent paradox, we assayed the intracellular FA concentration of cells inoculated into MP media containing 3.5mM succinate and 5mM FA over a 12 h period. We observed increasing intracellular FA concentrations for the first 6 h in all strains (**Fig 6**). Intriguingly, FA concentrations decreased dramatically after 6 h in strains lacking *efgA*, reaching intracellular concentrations of 0.5-1.0 mM after 12 h, ~ four-fold lower than WT, which sustained intracellular concentrations >4 mM for the remaining 6 h of the experiment. Additionally, we observed that strains lacking *ttmR* (*ΔttmR, ΔefgA ΔttmR*) had modestly lowered intracellular FA compared with cells encoding functional *ttmR* at all timepoints. Together, these results combined with previous work [22] suggest that EfgA-mediated translational arrest prevents cells from detoxifying internally accumulated FA during exogenous FA shock and that TtmR-mediated transcriptional regulation leads to modestly higher intracellular exposure to exogenous FA, possibly due to less FA entering the cells.

### Metabolic imbalance shifts towards formate and minimizes formaldehyde imbalance in the presence of lanthanides

Methylotrophic metabolisms tend to be mediated by multiple MeOH dehydrogenases that are regulated by the presence of lanthanide metals [32,33]. In *M. extorquens* PA1, lanthanides activate the expression of the alternative lanthanide-dependent MeOH dehydrogenase XoxF and turns off expression of MxaFI [32,33]. To determine the mechanisms of FA homeostasis at play in lanthanide-based physiology, we repeated the formate to MeOH carbon switch experiments with lanthanide-supplemented media.

**Fig 7:**
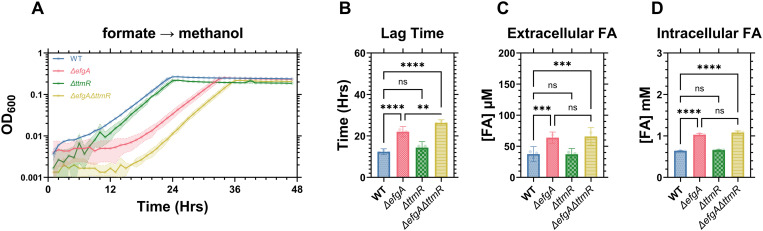
The presence of lanthanum minimizes the lag phase and FA imbalance during the transition from formate to MeOH. A) Growth of *M. extorquens* PA1 strains (WT, blue; Δ*efgA*, red; *ΔttmR*, green; *ΔefgA ΔttmR*, yellow) during carbon source transition from formate to MeOH with 2 µM lanthanum. B) Lag times of each strain. C) External FA concentration at the end of lag phase of each strain. D) Intracellular FA concentration of each strain. Error bars represent the 95% confidence interval of three biological replicates. Statistical significance was determined using a Brown-Forsythe and Welch’s ANOVA. * = p < 0.05, ** = p < 0.01, *** = p < 0.001.

**Fig 8:**
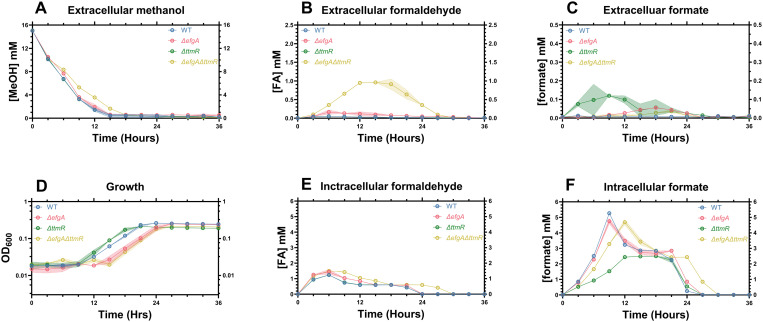
Metabolite imbalance is shifted towards formate in the presence of lanthanides in strains that cannot maintain FA homeostasis. Concentration of extracellular MeOH (A), extracellular formaldehyde (B), extracellular formate (C), optical density (D), intracellular formaldehyde (E), intracellular formate (F) and viability (G) of *M. extorquens* PA1 (WT, blue; *ΔefgA*, red; *ΔttmR* [green; *ΔefgA ΔttmR* [yellow]) cultures. Error shading represents the 95% confidence interval of the metabolite concentration of three independent biological replicates measured in technical triplicate. Peak metabolite values and associated statistical analysis can be found in S5 Table.

In WT, the transition from formate to MeOH in lanthanide-containing medium resulted in subdued FA imbalance (maxima: extracellular levels = 40 µM; intracellular levels = 0.6 mM) compared to lanthanide-free conditions (maxima: extracellular levels = 100 µM; intracellular levels = 1.8 mM) without a substantial effect on the lag time (**Figs 7**, S6). Interestingly, although the peak extracellular formate concentrations were also decreased compared to the no lanthanide condition (10 µM vs. 125 µM), the internal formate levels were notably higher with lanthanides (peak = 5–6 mM; steady state = 2.5-3 mM) versus without (peak = 2.5 mM; steady state = 1.8 mM) (**Figs 4F**, **8F**, S3B, S6B). Therefore, cells experience less FA imbalance in the presence of lanthanides, leading to a more efficient formate to MeOH transition.

When the *ΔefgA* mutant transitioned from formate to MeOH with lanthanum present, it had increased intracellular and extracellular FA concentrations and a lag phase defect compared to WT. Notably, the FA imbalance was more minor than the no lanthanide condition, consistent with lanthanides lowering intracellular FA levels, resulting in a less severe lag defect (**Figs 7C**, **7D and** S6C). Additionally, there was a minor increase in extracellular formate in the *ΔefgA* mutant (**Figs 8C**, S6C). In contrast to the *ΔefgA* mutant, in the *ΔttmR* mutant, the duration of lag phase and the extracellular and intracellular FA concentrations are statistically indistinguishable from WT, though the lag phase defect was not completely rescued (**Fig 7**). Consistent with this, the lag phase and extracellular and intracellular FA concentrations in the *ΔefgA ΔttmR* strain were each identical to that of the *ΔefgA* mutant apart from a minor, but statistically significant, exacerbation of the lag phase defect (**Fig 7**). Compared to WT, the *ΔttmR* mutant had increased extracellular formate and decreased intracellular formate, suggesting that the *ΔttmR* strains loses some formate to the extracellular environment during the transition (**Figs 8C**, **8F**, S6E **and** S6F).

Collectively, these data demonstrate that lanthanide-based physiology reduces FA imbalance during transitions to MeOH utilization. Further, they elucidated that *efgA* is the first line of defense required to maintain cellular FA homeostasis (**Fig 7D**), that the role of *ttmR* is secondary (**Fig 7**A-C), and that both contribute to FA homeostasis independent of the presence of lanthanides. Lastly, these results show that in strains unable to maintain FA homeostasis during the transition to MeOH, the metabolic imbalance is shifted from FA towards formate in the presence of lanthanides, likely alleviating FA-induced stress.

### ExaF promotes FA homeostasis and contributes to the optimal transition to lanthanide-mediated MeOH utilization

Previous work established that the lanthanide-dependent ethanol dehydrogenase ExaF can mitigate FA toxicity, likely via FA oxidation [23]. Given that the presence of lanthanides lowered extracellular FA concentrations in all strains and raised intracellular formate concentrations in most (not in the *ΔttmR* mutant, which loses formate to the environment), we hypothesized that ExaF was converting FA to formate *in vivo*. To test this, we generated markerless deletions of *exaF* in WT and the three mutant backgrounds. For comparison to cells that had lost their ability to sense and utilize lanthanides, we also generated analogous mutants lacking *lutH*, which encodes the exclusive lanthanide transporter [34,35]. We conducted a formate to MeOH carbon transition experiment with and without lanthanides present, and quantified growth, lag phase duration, and both extracellular and intracellular FA concentrations.

**Fig 9:**
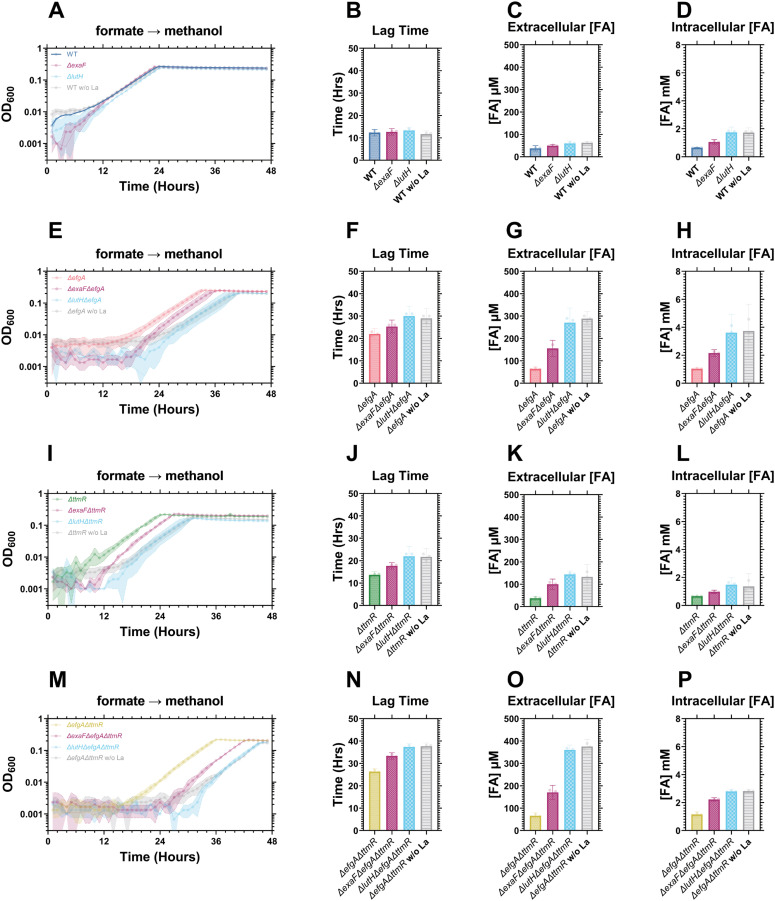
ExaF contributes to FA homeostasis by lowering the FA concentration. A,E,I,M: Growth of *M. extorquens* PA1 strains (WT, blue; *ΔefgA*, red; *ΔttmR*, green; *ΔefgA ΔttmR*, yellow) and corresponding derivatives (*ΔexaF*, purple; *ΔlutH*; sky blue) during carbon source transition from formate to MeOH. B,F,J,N: Lag times. C,G,K,O: Extracellular FA concentration at the end of lag phase. D,H,L,P: Intracellular FA concentration. Error bars represent the 95% confidence interval of three biological replicates. Statistical significance was determined using a Brown-Forsythe and Welch’s ANOVA. * = p < 0.05, ** = p < 0.01, *** = p < 0.001.

When lanthanides were absent, strains lacking *exaF* or *lutH* had no observable changes relative to the parent strains in growth, lag phase duration, extracellular FA concentration, or intracellular FA concentrations. Thus, confirming that neither *exaF* nor *lutH* play a role in maintaining FA homeostasis without lanthanides (S7 Fig). With lanthanides present, we observed analogous phenotypic trends across all genetic backgrounds (**Fig 9**). The loss of *exaF* increased extracellular and intracellular FA concentrations and the duration of the apparent lag phase (except in WT) (**Fig 9A**, **9B**). When we deleted *lutH*, these phenotypes were indistinguishable from the parent strains in the absence of lanthanides (**Fig 9**). Thus, it appears that ExaF contributes to maintaining FA homeostasis during the transition to MeOH in the presence of lanthanides, but that activation of ExaF is not the only lanthanide-induced physiological change required for maintaining FA homeostasis.

## Discussion

Herein, we demonstrated that EfgA and TtmR play independent, but complementary, roles in maintaining FA homeostasis during transitions to FA-generating carbon sources. The novel FA sensor EfgA plays the primary role in mediating homeostasis by coupling FA sensing to translation inhibition during transient FA imbalance to prevent cell death. The regulatory protein TtmR plays a secondary role in maintaining C_1_ homeostasis and utilization by mediating release of obligate C_1_ intermediates, including FA, to the extracellular space. Further, in the presence of lanthanides, ExaF converts excess FA into formate, which in turn minimizes need for TtmR and EfgA. Our overall model for FA homeostasis represents a novel combination of strategies to cope with a toxic metabolite that has broad implications for how *M. extorquens* survives and competes in its native environment and for biotechnological applications of native and synthetic methylotrophs. The combinatorial nature of this model is emblematic of homeostasis generally, as homeostatic regulation is not merely the product of a single negative feedback cycle but reflects the complex interaction of multiple feedback systems that can be modified by higher control centers.

**Fig 10:**
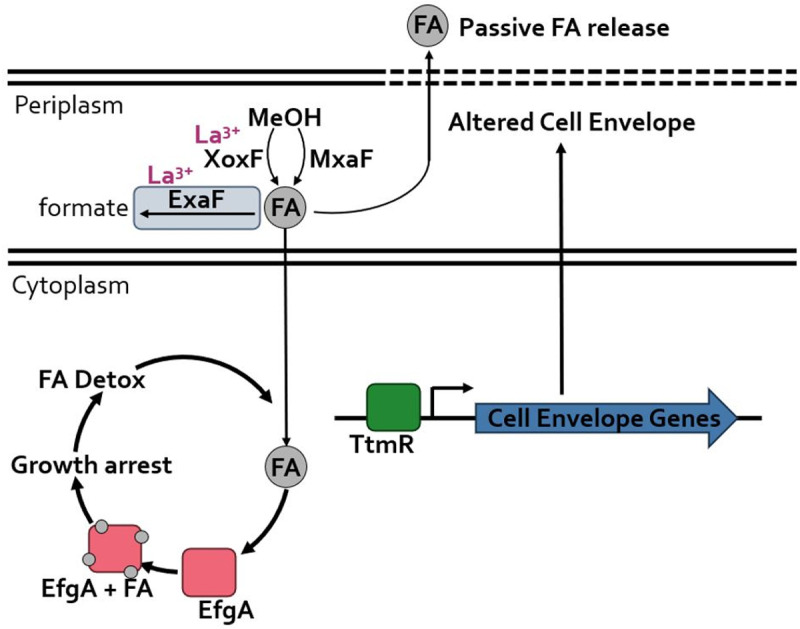
Model of FA Homeostasis: Formaldehyde (FA) can be produced by either MxaF or XoxF depending on the presence of lanthanides. In the presence of lanthanides, ExaF contributes to periplasmic FA detoxification by oxidizing FA to formate. EfgA senses the FA concentration and inhibits translation leading to growth arrest, allowing FA to be detoxified while preventing cell death. TtmR regulates the expression of cell envelope genes leading to alterations in cell envelope that change the cells permeability to FA.

During carbon source transitions where FA naïve cells encounter high fluxes of FA-generating growth substrates such as MeOH, cells are transiently FA-imbalanced, releasing FA into the growth medium. In strains lacking *efgA* or *ttmR*, this imbalance is exacerbated, resulting in a further increase in FA released and an apparent lag defect. In an *efgA* mutant, the observed correlation between the concentrations of extracellular FA and the lag phase duration reflected elevated intracellular FA concentrations. Unexpectedly, there was no correlation between extracellular and intracellular FA in the *ΔttmR* mutant. Despite also sharing a lag phase defect and increased extracellular FA, there was no difference in its intracellular FA concentrations (**Fig 4E**). Unlike in the *efgA* mutant, there was no substantial pause in MeOH consumption, nor any decrease in viability that would suggest FA-induced toxicity (**Figs 4E**, **5**). This is consistent with *ttmR* playing a secondary role in FA homeostasis and indicative of *efgA* being sufficient and necessary to maintain intracellular FA homeostasis.

Though intracellular FA was unchanged in the *ttmR* mutant, intracellular formate was decreased (**Fig 4C**). This was unexpected given that prior transcriptomic analyses of the *ΔttmR* mutant showed that during growth on MeOH, two formate dehydrogenases (*fdh2*, *fdh4*) and the formate assimilation pathway (*ftfL*, *fch, mtdA*) were modestly, but significantly, decreased in expression and one might have predicted that intracellular formate pools would be inflated [27]. Herein, we observed similar trends of decreased expression by real-time qPCR for *mxaF, ftfL, mtdB* and *hprA*, genes that represent different portions of methanol utilization pathways (S5 Fig). We also observed upregulation of *fdh3* which localizes to the periplasm and may draw down the intracellular formate concentration or partially prevent release of formate to the extracellular space. In addition to accumulating extracellular FA, we showed that the *ΔttmR* mutant accumulated considerable extracellular formate (~2 mM at its peak, **Fig 4C**). We predict that the metabolite loss experienced by the *ΔttmR* mutant prevents cells from entering log phase until enough extracellular metabolites build up to shift equilibrium towards metabolite uptake by diffusion. Indeed, our results suggest that extracellular metabolite concentrations for both FA and formate peak at approximately the same time, near when the *ΔttmR* strain enters log phase (**Fig 4C**, **4D**). Our data suggest that the lag phase defect observed in the *ΔttmR* mutant may be a direct result of formate loss and failure of cells to reach sufficient intracellular steady-state concentrations.

Given that there is no energy gradient across the outer membrane in Gram-negative bacteria, and the periplasm lacks ATP, the loss of metabolites to the environment is likely passive and mediated by diffusion. This suggests that either the outer membrane is altered in the *ΔttmR* mutant such that it is more permeable and prone to metabolite loss and/or the inner membrane has altered permeability which inhibits efficient transfer of metabolites into the cytoplasm. Our work confirmed the altered expression pattern of genes involved in LPS and O-antigen synthesis during the formate to MeOH transition. During growth, these cell envelope changes may effectively dilute the cellular FA to non-toxic levels by allowing release of some FA to the extracellular space, suggesting that finely tuned expression of *ttmR* can allow cells to mediate metabolite offloading/retention. In contrast to the suggestion of a more permeable outer membrane, the Δ*ttmR* mutant experienced decreased internal FA levels upon exogenous FA treatment, which is likely a contributing factor to its increased FA resistance (**Fig 6**). Although this may represent a difference in membrane sidedness that differentially impacts FA permeability, it is also possible that this was due to differences in growth conditions, or in TtmR-mediated changes in the cellular rate of FA detoxification or the composition of the inner membrane of the cell. Future work will address details of how TtmR impacts each of the cell membranes under both exogenous FA treatment, where FA is applied as a stressor and not a sole carbon source, and under a transition to methylotrophy, where all carbon must be funneled through FA and heightened loss of this molecular pool may result in delays to growth.

A unique and seemingly contradictory aspect of the model of homeostasis we present in this study lies in the differential response of the ∆*efgA* strain to exogenously added versus endogenously generated FA. In observing internal levels of FA that were heightened in cells lacking *efgA* relative to other strains in the transition from formate to MeOH, it became apparent that this was a source of cell death. However, tracking internal levels of FA under exogenously added FA conditions demonstrated that the ∆*efgA* strain is better suited to mitigate this source of FA than the wild-type strain. Though unexpected, this result is consistent with the fact that ∆*efgA* cells continue translation even in the face of heightened FA conditions, and thus likely allow for the detoxification of this heightened FA stress. Additionally, periplasmically generated FA might have more rapid access to the interior of the cell than exogenous FA, leading to a higher risk of FA imbalance, damage, and toxicity. In contrast, the ∆*ttmR* mutant experienced intracellular FA concentrations more similar to wild-type throughout the 12 h FA shock experiment, as it did during the formate to methanol transition experiments presented herein. Combined, these data emphasize the central role of EfgA in maintaining intracellular FA levels and suggest that the ∆*ttmR* strain possesses a distinct mechanism for increased resistance to exogenous FA apart from that of the ∆*efgA* strain. In previous transcriptomic analyses, we found that gene expression patterns of the ∆*ttmR* mutant closely resembled those of a FA-tolerant subpopulation which showed overexpression of genes encoding universal stress proteins and heat shock proteins [27]. Based on these findings and the current data, we hypothesize that the FA resistance of the ∆*ttmR* mutant is not the result of increased FA oxidation that reduces FA exposure; rather, it is achieved via direct mitigation of damage caused by elevated intracellular FA and potentially diminished FA exposure via alterations to intrinsic cellular barriers.

Finally, the presence of lanthanides activates *xoxF* and *exaF* to aid in the catabolism and detoxification of C_1_ compounds, respectively. ExaF, a periplasmic alcohol dehydrogenase, catalyzes the first step in ethanol utilization [36] and has been demonstrated to catalyze the oxidation of FA to formate *in vitro*, and reduce FA toxicity and FA levels *in vivo* [23]. Overall, ExaF-mediated catalysis shifted C_1_ metabolic pools, lowering FA concentrations while increasing formate concentrations. This excess formate may explain the previously noted upregulation of *fdh4* in lanthanide-containing conditions and demonstrates that formate pools are sufficiently high to promote assimilation. Our data suggest that ExaF converts FA to formate *in vivo* and thus serves as an alternate FA oxidation system that localizes some cellular FA detoxification to the periplasm, adding a layer of FA stress mitigation strategies to the identified roles of EfgA and TtmR, and expanding our understanding of where FA detoxification occurs.

Our combined results allowed us to construct an overall model of FA homeostasis in *M. extorquens* PA1 (**Fig 10**). Initially, upon exposure to MeOH, cells begin to generate FA, eventually causing an internal concentration spike, which is primarily sensed in the cell via direct binding of EfgA to cytoplasmic FA [22]. FA-bound EfgA then assumes an active conformation, leading to a cell-wide stalling in translation and resulting in growth arrest. While cells are growth-arrested, FA concentrations are decreased to sub-lethal levels by oxidation to formate by the H_4_MPT pathway and/or ExaF, depending on the growth conditions. Once FA concentrations return to homeostatic levels, EfgA resumes its unbound form and translation resumes, allowing cells to resume growth [28]. In instances where FA concentrations are sufficiently high, TtmR secondarily becomes necessary for optimal growth.

Key features of the model rely on periplasmic compartmentalization of FA production. Compartmentalization is a common strategy to prevent damage by toxic metabolites, including toxic aldehydes. Few means of metabolite compartmentalization are appreciated in bacteria: they include proteinaceous microcompartments [37,38] and the recently discovered existence of membrane-less organelles [39,40]. We propose that the maintenance of FA homeostasis in *M. extorquens* leverages the periplasm as a membrane-separated compartment to mitigate FA toxicity. Interestingly, such compartmentalization is seemingly subpar, in that the toxic metabolite FA must enter the cytoplasm before detoxification occurs [24]. Here, we posit that by confining FA production to the periplasm, *M. extorquens* can employ additional, complementary strategies to mitigate FA toxicity: namely, local detoxification of excess FA by periplasmic alcohol dehydrogenases such as ExaF, and *ttmR-*based release of FA and other C_1_ metabolites to the extracellular space through cell envelope modulation. It is also a formal possibility that one mechanism that EfgA mediates is to effectively modulate the FA concentration gradient across the cytoplasmic membrane, and that EfgA and TtmR typically work together to coordinate periplasmic and cytoplasmic FA levels during transitions to FA-generating carbon sources to optimize metabolic flux and mitigate FA-induced toxicity. While intriguing to consider, such mechanistic work is beyond the scope of the work herein. Compartmentalization essentially gives *M. extorquens* several strategies to maintain FA homeostasis, including decreasing periplasmic concentration of FA through the utilization of lanthanide-dependent physiology, or the passive release of FA by altering the permeability of the cellular membranes.

The loss of FA to the environment may be detrimental to efficient MeOH utilization in batch culture. However, in some scenarios, this may provide a benefit. For example, having the ability to release FA into the environment when encountering FA concentration spikes due to sudden MeOH release by plants in the leaf environment could be beneficial by allowing cells to handle greater variability in MeOH availability, ultimately allowing them to capture more MeOH without the risk of toxifying the cytoplasm by overproducing FA. Such a phenomenon is likely threshold-dependent, meaning that releasing metabolites would only be beneficial when their concentrations reach levels that cause stress. In this way, the ability to release metabolites into the environment allows cells to strike a balance between optimizing carbon utilization and minimizing the internal accumulation of stressful reactive metabolites.

Together, this work represents a comprehensive analysis in characterizing strategies to maintain FA concentrations at sub-lethal homeostatic levels in a biological system. In the model methylotroph *M. extorquens*, which is necessarily adept in coordinating and navigating FA-dependent processes, FA homeostasis is enabled by two FA-responsive genes, *efgA* and *ttmR,* in conjunction with *exaF*, depending on the availability of lanthanides, and leverages compartmentalization of FA production. We describe how the deletion of either *efgA* or *ttmR* alters extracellular FA concentrations, manifesting growth defects during the transition to methylotrophy and FA-generating growth substrates in general. We show that in turn, disequilibrium of FA in both mutants results in more expansive metabolic consequences for the cell; specifically, FA toxicity in the *ΔefgA* mutant and carbon loss in the *ΔttmR* mutant. From this, we have constructed a model explaining the primary modes of FA homeostasis in *M. extorquens* PA1, and also have demonstrated that FA homeostasis is a complex and multi-layered process in this model methylotroph, both in terms of involved genes and in terms of resulting physiological phenotypes.

## Materials and methods

### Bacterial strains, media, and chemicals

Bacterial strains used in this study (S1 Table) are derived from *Methylobacterium extorquens* PA [41] with cellulose synthase genes deleted (*ΔbcsABZ*) to prevent aggregation and optimize growth measurements in liquid culture [42]. Bacterial strains were cultivated using *Methylobacterium* piparazine-N,N’-bis(2-ethanesulfonic acid) (MP) medium [42] with 3.5 mM succinate; 15 mM MeOH, 20 mM acetate, 20 mM oxalate, 20 mM formate, or 20 mM methylamine as a sole source of carbon and energy. When grown on solid MP medium (15 g/L Bacto agar), 15 mM succinate was provided as the sole carbon. Chemicals and reagents were purchased from Sigma Aldrich.

### Genetic approaches

Markerless deletions were generated by allelic exchange as previously described with modifications [43,44]. Bi-parental conjugations were performed by mixing *E. coli* S17-1 cells carrying the pPS04-based or pLW18-based donor plasmid with *M. extorquens* PA1 (S2 Table). The mixture was grown overnight on nutrient agar plates at 30 °C, resuspended in MP medium lacking carbon and nitrogen, serially diluted, and plated on selective medium supplemented with 15 mM succinate, 5 mM methylamine (as the sole source of nitrogen for counterselection against *E. coli*), and 50 μg/mL kanamycin for pPS04-based or 50 μg/mL tetracycline for pLW18-based plasmids. Sucrose selection for pPS04 or pLW18 mediated allelic exchange was accomplished by streaking isolated colonies from selection plates on MP medium supplemented with 15 mM succinate and 5% sucrose. Donor plasmids and primers were designed using SnapGene software. Plasmids were assembled using New England Biolabs (NEB) HiFi assembly kits.

### Growth quantification

Growth of *M. extorquens* PA1 was quantified as previously described [44]. Starter cultures grown in MP medium with 3.5 mM succinate from isolated colonies. Duration of acclimation cultures was determined for each carbon source used (~30 h: succinate, ~ 40 h: MeOH, ~ 60 h: oxalate, acetate, formate). Growth was quantified by measuring optical density at 600 nm in 48-well polystyrene plates (Falcon, Ref No. 351178) sealed with adhesive optical film (VWR, Cat No. 60941–064) to prevent evaporation of volatile metabolites at 30 °C using a BioTek Epoch 2.

### Determination of the end of lag phase

The time point representing the end of lag phase was determined as the first time point at which the non-linear regression line of three consecutive readings was statistically indistinguishable from the non-linear regression line of mid-log phase cells.

### Methanol quantification

MeOH concentrations in culture media were measured using a coupled colorimetric assay [45]. Briefly, cultures were centrifuged at 16,000 x *g* to pellet cells. A 10 μL sample of supernatant was diluted 1:10 in MP medium without carbon or nitrogen. In a dark room a 10 μL sample was then added to 90 μL of reaction master mix (25 U/mL yeast alcohol oxidase, 15U/mL horseradish peroxidase, 1 mg/mL 2,2’-azino-bis(3-ethylbenzothiozoline-6-sulfonic acid) diammonium salt in MP medium without carbon or nitrogen. The reaction was incubated for 5 min at room temperature in the dark before absorbance readings were taken at 420 nm on a SpectraMax i3x spectrophotometer. MeOH standards (50, 100, 500 nM; 1, 5, 10, 50, 100, 500 µM; 1, 5 mM) were prepared from MP medium with 15 mM MeOH stock and used to generate a standard curve alongside all unknown sample measurements.

### Extracellular formaldehyde quantification

Formaldehyde (FA) concentrations in culture media were measured using the colorimetric Nash method as described previously [46]. Briefly, 1000 μL aliquots from cultures were centrifuged at 16,000 x *g* to pellet cells. Next, 180 μL of the resulting supernatant was combined with 20μL of Nash reagent B (2 M ammonium acetate, 50 mM glacial acetic acid, 20 mM acetylacetone) in 96-well polystyrene plates in technical triplicate. Reaction plates were incubated for 10 min at 60 °C and cooled to room temp before reading absorbance at 432 nm on a SpectraMax i3x spectrophotometer. FA standards (2.5, 5, 10, 25, 50, 100, 250, 500 µM; 1, 2.5 mM) were prepared from 1 M FA stock solutions and used to generate a standard curve alongside all unknown sample measurements.

### Intracellular formaldehyde quantification

Intracellular FA concentrations were measured using the colorimetric Purpald method and the internal cell volume was calculated as described previously [22,23]. Briefly, 5mL aliquots from cultures were centrifuged at 16,000 x *g* to pellet cells. Supernatants were removed, and pellets were resuspended in 25 mM Tris-HCl pH 8.0 150mM NaCl. Cells were lysed by 5 rounds of bead beating on a Biospec Mini Bead Beater 24 for 1 minute followed by 2 min on ice. Cell debris was removed by centrifugation at 16,000 x *g*. A 50 μL of sample was incubated with 50 μL of 34 mM Purpald reagent for 20 min in 96 well polystyrene plates in technical triplicate. The reaction was stopped with 33 mM sodium periodate and absorbance was read at 550 nm on a SpectraMax i3x spectrophotometer. FA standards (0.1, 0.25, 0.5, 1, 2.5, 5, 10, 25, 50, 100 µM) were prepared daily from 1 M FA stock solutions and used to generate a standard curve alongside all unknown sample measurements.

### Formate quantification

Formate concentrations were measured using a yeast formate dehydrogenase assay as described previously [47]. A 10 μL of sample was added to 90 μL of the reaction master mix (2 U/mL yeast formate dehydrogenase, 2 mM NAD^+^ in 1X PBS) in 96-well polystyrene plates. Samples were incubated at 37 °C for 1 h before reading absorbance at 340 nm on a SpectraMax i3x spectrophotometer. Formate standards (1, 2.5, 5, 10, 25, 50, 100, 250, 500, 1000 µM) were prepared from a 1 M sodium formate stock solution. For extracellular samples, supernatant was aliquoted after removal of cells by centrifugation at 16,000 x *g.* For intracellular samples, cell lysates were prepared, and internal cell volume was calculated as described above in *Intracellular FA Quantification*.

### Viability quantification

Viability was quantified using the BacTiter-Glo Microbial Cell Viability Assay kit (Promega, Cat No. G8232) according to the manufacturer’s protocol, after validating its reliability by comparing growth measured by absorbance and CFU plating (S8 Fig). Briefly, 100 μL of cell cultures was added to 100 μL of the BacTiter-Glo working reagent. The mixture was briefly vortexed to mix and was incubated at room temperature for 5 min before luminescence was measured on a SpectraMax i3x spectrophotometer with a 100 μs integration time.

### Real-time quantitative PCR

RT-qPCR was conducted as previously described [44]. *M. extorquens* PA1 cultures were harvest by centrifugation in mid-log phase (OD600 0.30 + /- 0.02). RNA was extracted using the RNA Clean and Concentrator kit from Zymo Research. Genomic DNA was removed by treating the RNA with DNase I following the manufacturer’s instructions (ThermoFisher). cDNA was synthesized using the High-Capacity cDNA Reverse Transcription kit from Applied Biosystems, following the manufacturer’s protocol. qPCR analysis was conducted in a 10 µL reaction volume in Fast Optical 96-Well reaction plates (MicroAmp) using the Maxima SYBR Green/ROX qPCR Master Mix (ThermoFisher) on a StepOnePlus Real-Time PCR system (Applied Biosystems). Each qPCR reaction was performed in triplicate for three independent biological replicates. StepOne Software v2.3 was utilized to acquire and analyze the qPCR data and cycle threshold (CT) values were calculated for each reaction. The technical triplicate values were averaged, normalized to *recA*, and analyzed using the 2-ΔΔCt method [48]. Primers used for qPCR analysis are listed in S3 Table.

## Supporting information

S1 Fig Workflow diagram of experiments: Workflow diagram depicting experimental set up of metabolic transition experiments [A], and of metabolite tracking experiments during formate to methanol transitions [B]. (PDF)

S2 Fig No defect is observed when transitioning to single carbon substrates that do not generate FA. A,C,E,G) Growth of *M. extorquens* PA1 strains (WT, blue; *ΔefgA*, red; *ΔttmR*, green; *ΔefgA ΔttmR*, yellow) when transitioning from growth on succinate to growth on formate [A], methylamine [C], acetate [E] or oxalate [G] B,D,F,H) Lag times of *M. extorquens* PA1 strains. Error bars represent the 95% confidence interval of three independent biological replicates.
(PDF)

S3 Fig Concentrations of C_1_ metabolites are imbalanced in strains that cannot maintain FA homeostasis during the transition from formate to MeOH. Concentration of MeOH, dark blue; FA, green; and formate, red measured in the supernatant (A-D) or intracellularly (E-H) of *M. extorquens* PA1 (WT [A,E], *ΔefgA* [B,F], *ΔttmR* [C,G], *ΔefgA ΔttmR* [D,H]) cultures. OD_600_ measurements are shown independent of axis in light gray for comparison to growth phase during the experiment. Error shading represents the 95% confidence interval of the metabolite concentration of three independent biological replicates measured in technical triplicate. Peak metabolite values and associated statistical analysis can be found in S4 Table.
(PDF)

S4 Fig Intracellular formaldehyde is elevated in strains lacking *efgA* during metabolic transitions to methanol. Intracellular formaldehyde concentrations measured by purpald assay in *M. extorquens* PA1 (WT, blue; *ΔefgA*, red; *ΔttmR*, green; *ΔefgA ΔttmR*, yellow) during metabolic transitions to methanol.
(PDF)

S5 Fig LPS and O-antigen synthesis genes are dysregulated in strains lacking *ttmR.* Reverse-transcriptase quantitative PCR analysis of metabolic and LPS/O-antigen synthesis genes in *M. extorquens* PA1 strains (*ΔefgA*, red; *ΔttmR*, green; *ΔefgAΔttmR*, yellow) relative to WT. Bars represent the average of 3 biological replicates with each point representing the average of 3 technical replicates. Error bars represent the 95% confidence interval.
(PDF)

S6 Fig Metabolite imbalance is shifted towards formate in the presence of lanthanides in strains that cannot maintain FA homeostasis. Concentration of MeOH, dark blue; FA, green; and formate, red measured in the supernatant (A,C,E,G) or intracellularly (B,D,F,H) of *M. extorquens* PA1 (WT [A,B], *ΔefgA* [C,D], *ΔttmR* [E,F], *ΔefgA ΔttmR* [G,H]) cultures. OD_600_ patterns are shown independent of axis in light gray to contextualize the data with regard growth phase during the experiment. Error shading represents the 95% confidence interval of the metabolite concentration of three independent biological replicates measured in technical triplicate. Peak metabolite values and associated statistical analysis can be found in Table S5.
(PDF)

S7 Fig ExaF and LutH do not affect FA homeostasis in the absence of lanthanides. A,E,I,M: Growth of *M. extorquens* PA1 strains (WT, blue; *ΔefgA*, red; *ΔttmR*, green; *ΔefgA ΔttmR*, yellow) and corresponding derivatives (*ΔexaF*, purple; *ΔlutH*; sky blue) during carbon source transition from formate to MeOH. B,F,J,N: Lag times. C,G,K,O: Extracellular FA concentration at the end of lag phase. D,H,L,P: Intracellular FA concentration. Error bars represent the 95% confidence interval of three biological replicates. Statistical significance was determined using a Brown-Forsythe and Welch’s ANOVA. No significant differences were determined.
(PDF)

S8 Fig BacTiter-Glo recapitulates death and growth phenotypes of formaldehyde treated cells. A) Relative luminescence of cells as determined using the BacTiter-Glo kit. B) Viable colony forming units as determined by dilution plating. C) Optical density of cultures. Untreated cells (Blue), FA treated cells (Red). Error shading represents the 95% confidence interval.
(PDF)

S1 Table Strains used in this study. (XLSX)

S2 Table Plasmids used in this study. (XLSX)

S3 Table Primers used in this study. (XLSX)

S4 Table Metabolite concentrations and statistics for data displayed in Fig 4. Statistical significance was determined using a Brown-Forsythe and Welch’s ANOVA. * p < 0.05, ** p < 0.01, *** p < 0.001, **** p < 0.0001.
(XLSX)

S5 Table Metabolite concentrations and statistics for data displayed in Fig 8. Statistical significance was determined using a Brown-Forsythe and Welch’s ANOVA. * p < 0.05, ** p < 0.01, *** p < 0.001, **** p < 0.0001.
(XLSX)

S1 Data Data underlying the paper. (XLSX)
