# Supplementary material for: Methylobacterium extorquens PA1 utilizes multiple strategies to maintain formaldehyde homeostasis during methylotrophic growth
Source: PLoS Genet. 2025 Jun 9;21(6):e1011736. doi: 10.1371/journal.pgen.1011736 (PMC12180729; doi:10.1371/journal.pgen.1011736)
Supplement: S1 Fig — (PDF) [file pgen.1011736.s001.pdf]

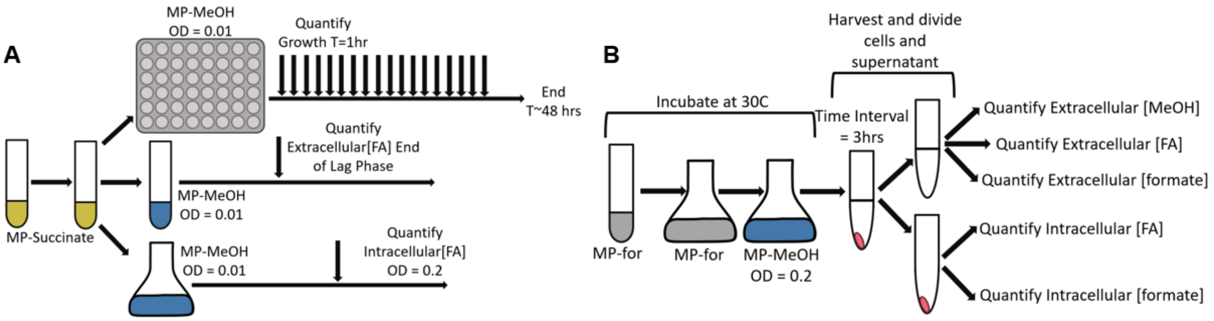

S1 Fig. Workflow diagram of experiments: Workflow diagram depicting experimental set up of metabolic transition experiments [A], and of metabolite tracking experiments during formate to methanol transitions [B].
