# Supplementary material for: Methylobacterium extorquens PA1 utilizes multiple strategies to maintain formaldehyde homeostasis during methylotrophic growth
Source: PLoS Genet. 2025 Jun 9;21(6):e1011736. doi: 10.1371/journal.pgen.1011736 (PMC12180729; doi:10.1371/journal.pgen.1011736)
Supplement: S2 Fig — A,C,E,G) Growth of M. extorquens PA1 strains (WT, blue; ΔefgA, red; ΔttmR, green; ΔefgA ΔttmR, yellow) when transitioning from growth on succinate to growth on formate [A], methylamine [C], acetate [E] or oxalate [G] B,D,F,H) Lag times of M. extorquens PA1 strains. Error bars represent the 95% confidence interval of three independent biological replicates. (PDF) [file pgen.1011736.s002.pdf]

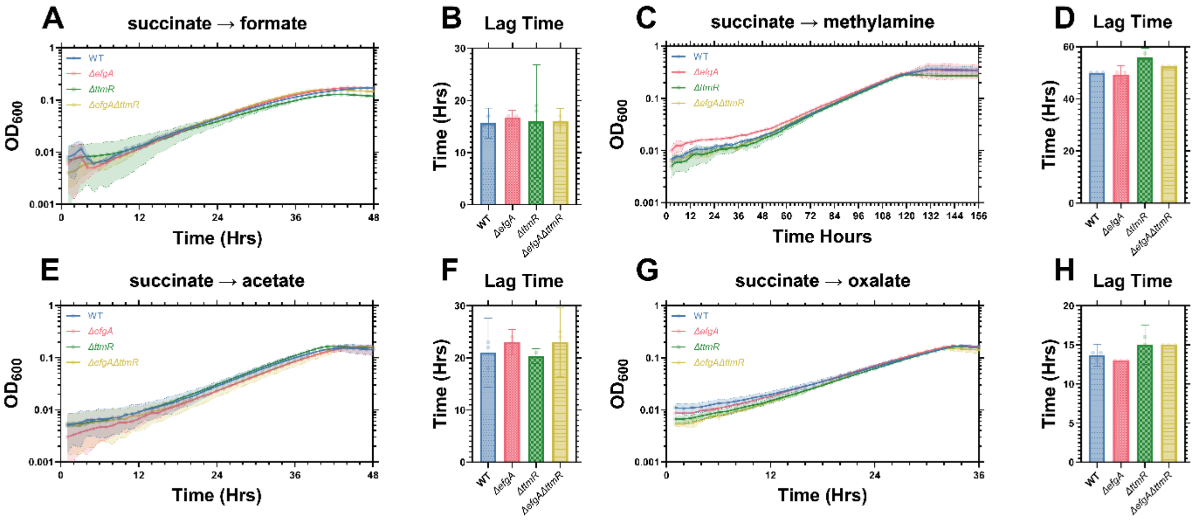

S2 Fig. No defect is observed when transitioning to single carbon substrates that do not generate FA. A,C,E,G) Growth of *M. extorquens* PA1 strains (WT, blue; *ΔefgA*, red; *ΔttmR*, green; *ΔefgA ΔttmR*, yellow) when transitioning from growth on succinate to growth on formate [A], methylamine [C], acetate [E] or oxalate [G]. B,D,F,H) Lag times of *M. extorquens* PA1 strains. Error bars represent the 95% confidence interval of three independent biological replicates.
