# Supplementary material for: Methylobacterium extorquens PA1 utilizes multiple strategies to maintain formaldehyde homeostasis during methylotrophic growth
Source: PLoS Genet. 2025 Jun 9;21(6):e1011736. doi: 10.1371/journal.pgen.1011736 (PMC12180729; doi:10.1371/journal.pgen.1011736)
Supplement: S3 Fig — Concentration of MeOH, dark blue; FA, green; and formate, red measured in the supernatant (A-D) or intracellularly (E-H) of M. extorquens PA1 (WT [A,E], ΔefgA [B,F], ΔttmR [C,G], ΔefgA ΔttmR [D,H]) cultures. OD600 measurements are shown independent of axis in light gray for comparison to growth phase during the experiment. Error shading represents the 95% confidence interval of the metabolite concentration of three independent biological replicates measured in technical triplicate. Peak metabolite values and associated statistical analysis can be found in S4 Table. (PDF) [file pgen.1011736.s003.pdf]

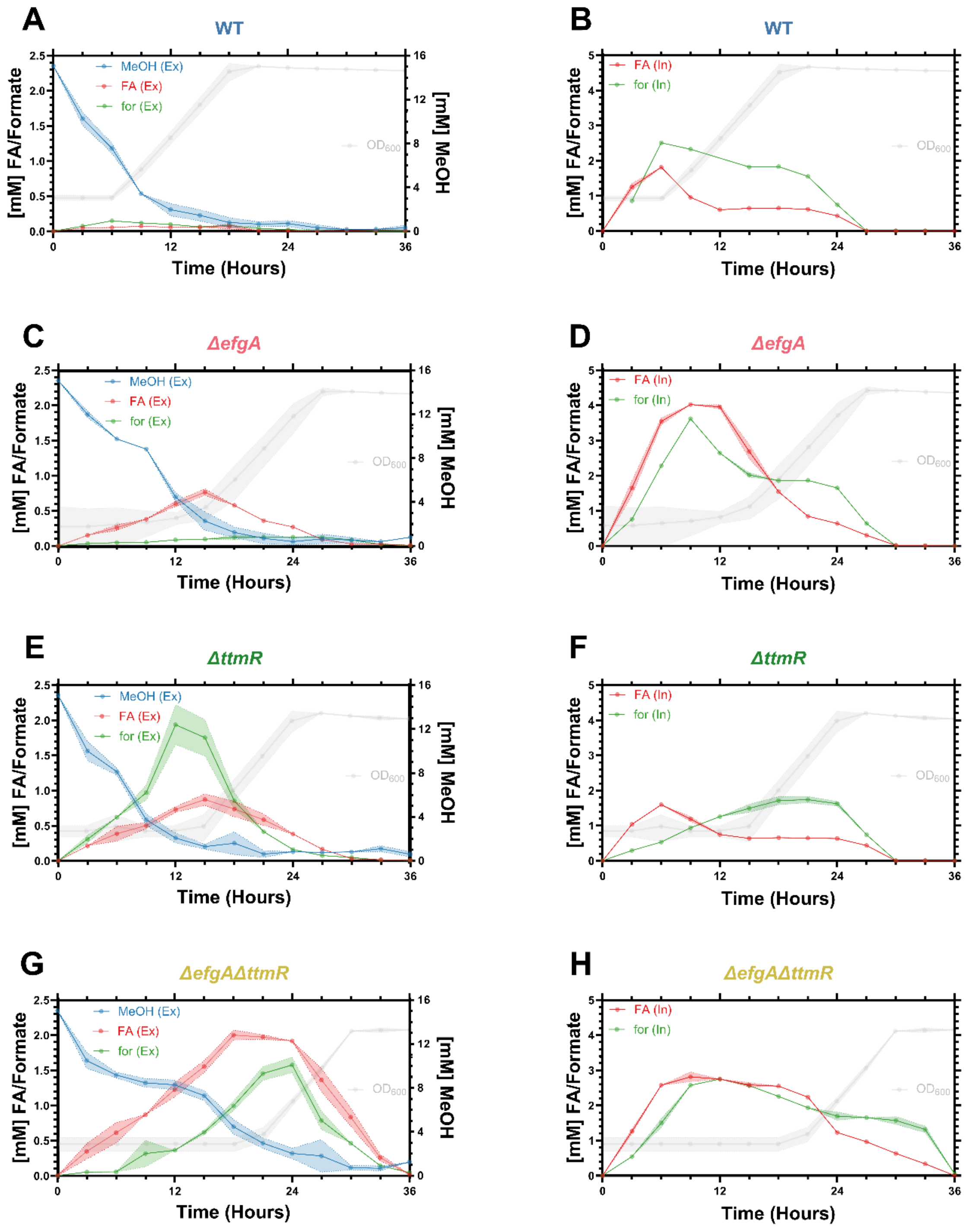

S3 Fig. Concentrations of C_1 metabolites are imbalanced in strains that cannot maintain FA homeostasis during transition to methylotrophy. Concentration of MeOH [dark blue], FA

[green], and formate [red] measured in the supernatant (A-D) or intracellularly (E-H) of *M. extorquens* PA1 (WT [A,E], *ΔefgA* [B,F], *ΔttmR* [C,G], *ΔefgA ΔttmR* [D,H]) cultures. OD_{600} measurements are shown independent of axis in light blue for comparison to growth phase during the experiment. Error shading represents the 95% confidence interval of the metabolite concentration of three independent biological replicates measured in technical triplicate. Peak metabolite values and associated statistical analysis can be found in **S4 Table.**
