# Supplementary material for: Methylobacterium extorquens PA1 utilizes multiple strategies to maintain formaldehyde homeostasis during methylotrophic growth
Source: PLoS Genet. 2025 Jun 9;21(6):e1011736. doi: 10.1371/journal.pgen.1011736 (PMC12180729; doi:10.1371/journal.pgen.1011736)
Supplement: S4 Fig — Intracellular formaldehyde concentrations measured by purpald assay in M. extorquens PA1 (WT, blue; ΔefgA, red; ΔttmR, green; ΔefgA ΔttmR, yellow) during metabolic transitions to methanol. (PDF) [file pgen.1011736.s004.pdf]

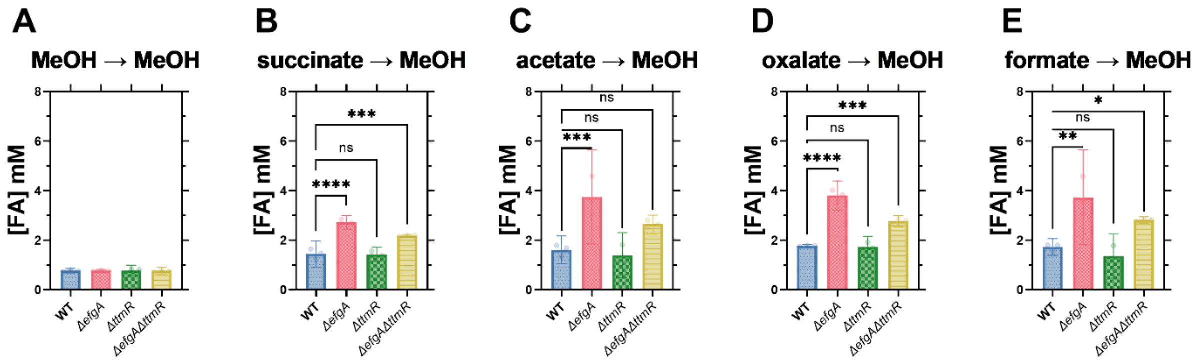

S4 Fig. Intracellular formaldehyde is elevated in strains lacking *efgA* during metabolic transitions to methanol. Intracellular formaldehyde concentrations measured by purpald assay in *M. extorquens* PA1 (WT, blue; *ΔefgA*, red; *ΔttmR*, green; *ΔefgA ΔttmR*, yellow) during metabolic transitions to methanol.
