# Supplementary material for: Methylobacterium extorquens PA1 utilizes multiple strategies to maintain formaldehyde homeostasis during methylotrophic growth
Source: PLoS Genet. 2025 Jun 9;21(6):e1011736. doi: 10.1371/journal.pgen.1011736 (PMC12180729; doi:10.1371/journal.pgen.1011736)
Supplement: S5 Fig — Reverse-transcriptase quantitative PCR analysis of metabolic and LPS/O-antigen synthesis genes in M. extorquens PA1 strains (ΔefgA, red; ΔttmR, green; ΔefgAΔttmR, yellow) relative to WT. Bars represent the average of 3 biological replicates with each point representing the average of 3 technical replicates. Error bars represent the 95% confidence interval. (PDF) [file pgen.1011736.s005.pdf]

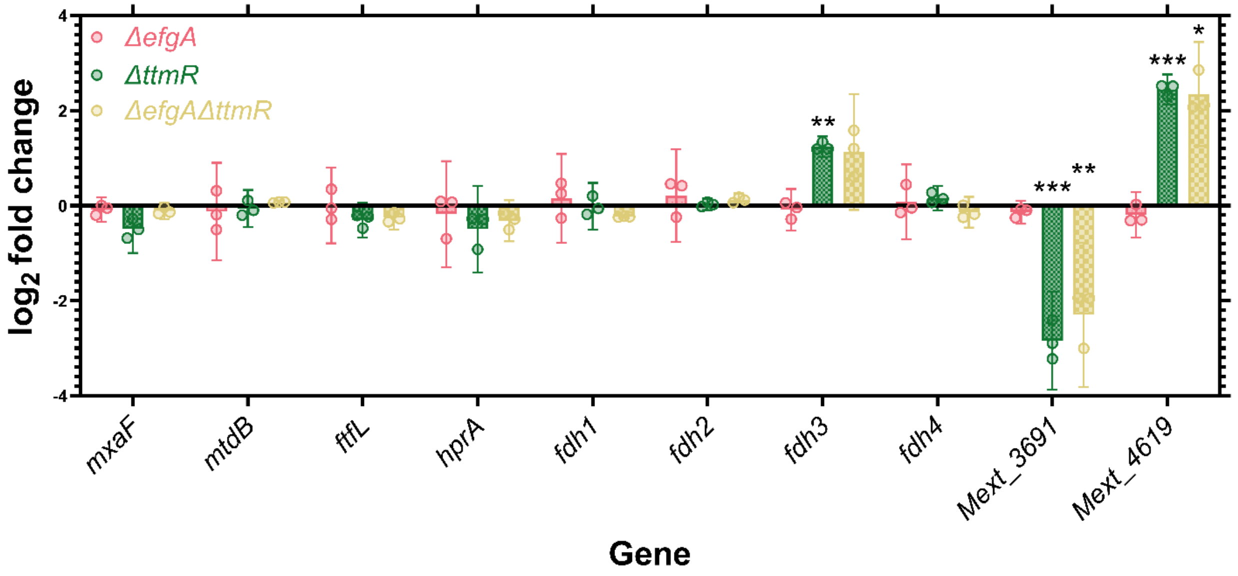

S5 Fig. LPS and O-antigen synthesis gens are dysregulated in strains lacking *ttmR*. Reverse-transcriptase quantitative PCR analysis of metabolic and LPS/O-antigen synthesis genes in *M. extorquens* PA1 strains (*ΔefgA* [red], *ΔttmR* [green], *ΔefgAΔttmR* [yellow]) relative to WT. Bars represent the average of 3 biological replicates with each point representing the average of 3 technical replicates. Error bars represent the 95% confidence interval.
