# Supplementary material for: Methylobacterium extorquens PA1 utilizes multiple strategies to maintain formaldehyde homeostasis during methylotrophic growth
Source: PLoS Genet. 2025 Jun 9;21(6):e1011736. doi: 10.1371/journal.pgen.1011736 (PMC12180729; doi:10.1371/journal.pgen.1011736)
Supplement: S6 Fig — Concentration of MeOH, dark blue; FA, green; and formate, red measured in the supernatant (A,C,E,G) or intracellularly (B,D,F,H) of M. extorquens PA1 (WT [A,B], ΔefgA [C,D], ΔttmR [E,F], ΔefgA ΔttmR [G,H]) cultures. OD600 patterns are shown independent of axis in light gray to contextualize the data with regard growth phase during the experiment. Error shading represents the 95% confidence interval of the metabolite concentration of three independent biological replicates measured in technical triplicate. Peak metabolite values and associated statistical analysis can be found in Table S5. (PDF) [file pgen.1011736.s006.pdf]

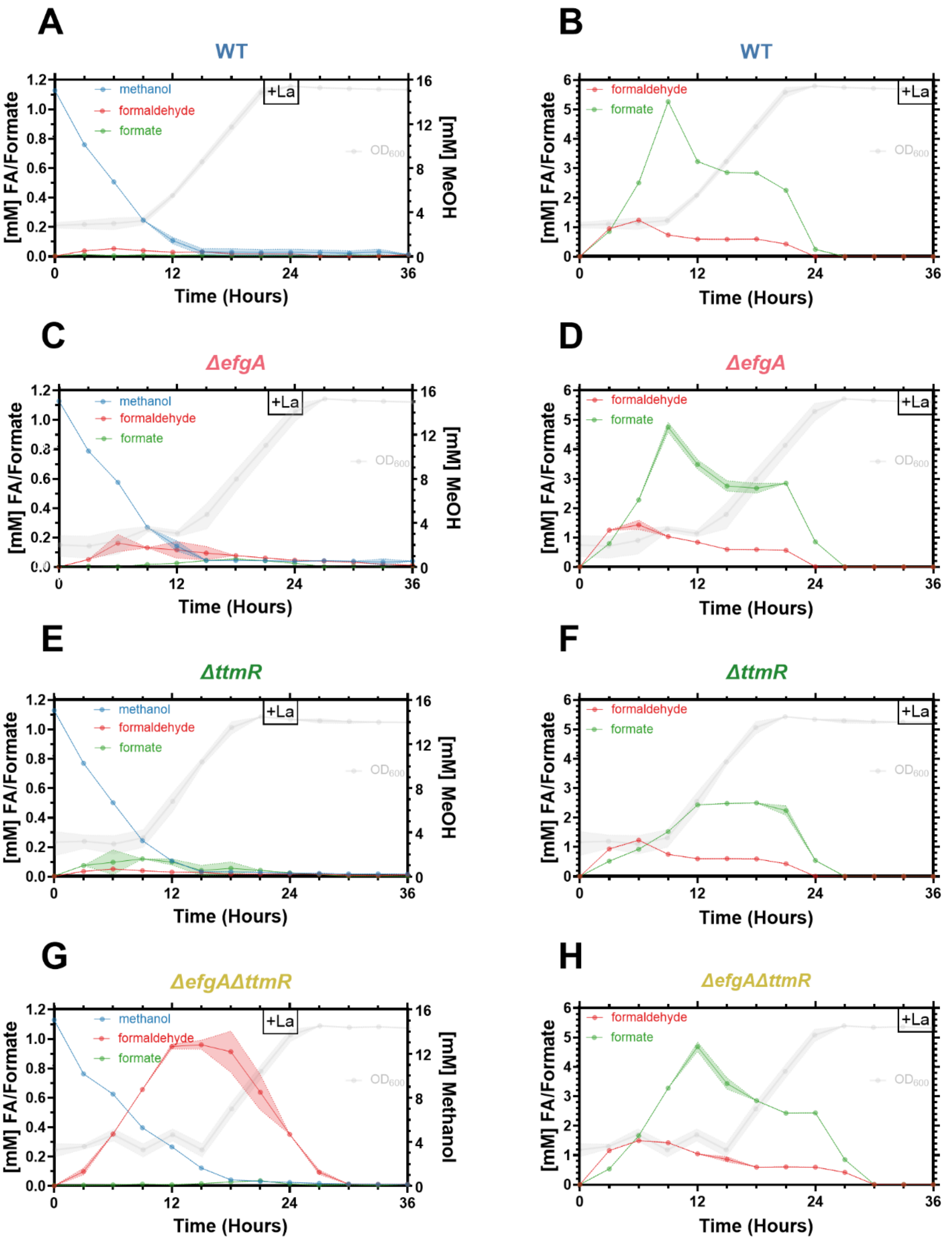

S6 Fig. Metabolite imbalance is shifted towards formate in the presence of lanthanides in strains that cannot maintain FA homeostasis. Concentration of MeOH [dark blue], FA [green],

and formate [red] measured in the supernatant (A,C,E,G) or intracellularly (B,D,F,H) of *M. extorquens* PA1 (wild-type [A,B], *ΔefgA* [C,D], *ΔttmR* [E,F], *ΔefgA ΔttmR* [G,H]) cultures. OD_{600} patterns are shown independent of axis in light gray to contextualize the data with regard growth phase during the experiment. Error shading represents the 95% confidence interval of the metabolite concentration of three independent biological replicates measured in technical triplicate. Peak metabolite values and associated statistical analysis can be found in **S5 Table.**
