# Supplementary material for: Methylobacterium extorquens PA1 utilizes multiple strategies to maintain formaldehyde homeostasis during methylotrophic growth
Source: PLoS Genet. 2025 Jun 9;21(6):e1011736. doi: 10.1371/journal.pgen.1011736 (PMC12180729; doi:10.1371/journal.pgen.1011736)
Supplement: S7 Fig — A,E,I,M: Growth of M. extorquens PA1 strains (WT, blue; ΔefgA, red; ΔttmR, green; ΔefgA ΔttmR, yellow) and corresponding derivatives (ΔexaF, purple; ΔlutH; sky blue) during carbon source transition from formate to MeOH. B,F,J,N: Lag times. C,G,K,O: Extracellular FA concentration at the end of lag phase. D,H,L,P: Intracellular FA concentration. Error bars represent the 95% confidence interval of three biological replicates. Statistical significance was determined using a Brown-Forsythe and Welch’s ANOVA. No significant differences were determined. (PDF) [file pgen.1011736.s007.pdf]

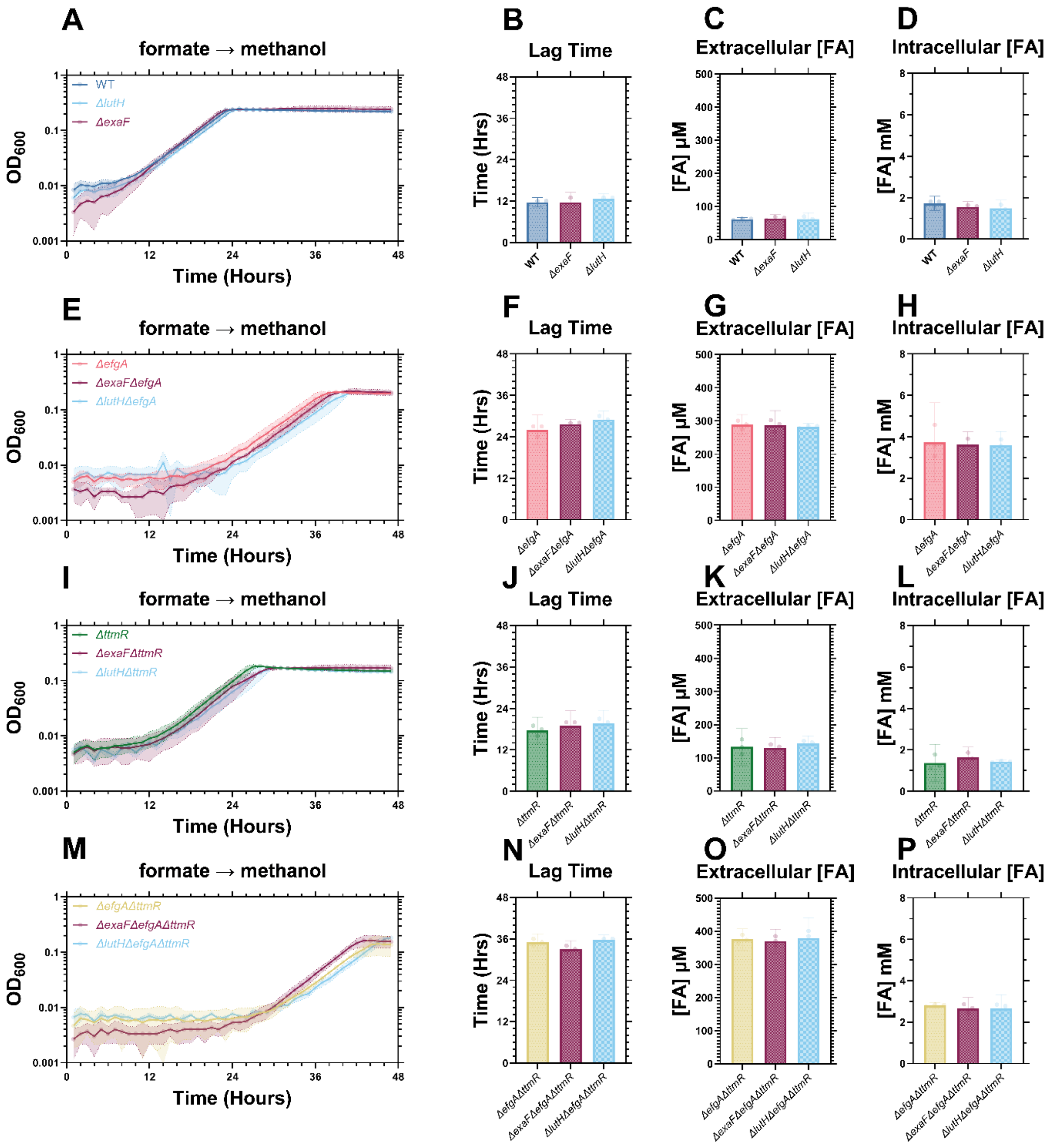

S7 Fig. ExaF and LutH do not affect FA homeostasis in the absence of lanthanides. A,E,I,M: Growth of *M. extorquens* PA1 strains (WT, blue; *ΔefgA*, red; *ΔttmR*, green; *ΔefgA ΔttmR*, yellow) (P) and corresponding derivatives (*ΔexaF*, purple; *ΔlutH*; sky blue) during carbon source transition from formate to MeOH. B,F,J,N: Lag times. C,G,K,O: Extracellular FA concentration at the end of lag phase. D,H,L,P: Intracellular FA concentration. Error bars

represent the 95% confidence interval of three biological replicates. Statistical significance was determined using a Brown-Forsythe and Welch's ANOVA. No significant differences were determined.
