# Supplementary material for: Methylobacterium extorquens PA1 utilizes multiple strategies to maintain formaldehyde homeostasis during methylotrophic growth
Source: PLoS Genet. 2025 Jun 9;21(6):e1011736. doi: 10.1371/journal.pgen.1011736 (PMC12180729; doi:10.1371/journal.pgen.1011736)
Supplement: S8 Fig — A) Relative luminescence of cells as determined using the BacTiter-Glo kit. B) Viable colony forming units as determined by dilution plating. C) Optical density of cultures. Untreated cells (Blue), FA treated cells (Red). Error shading represents the 95% confidence interval. (PDF) [file pgen.1011736.s008.pdf]

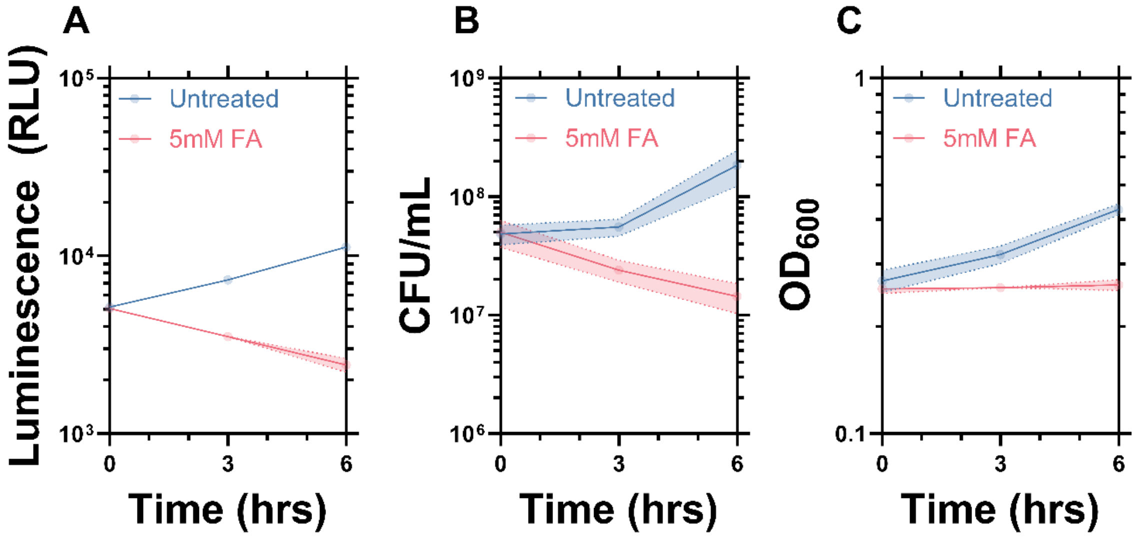

S8 Fig. BacTiter-Glo recapitulates death and growth phenotypes of formaldehyde treated cells. A) Relative luminescence of cells as determined using the BacTiter-Glo kit. B) Viable colony forming units as determined by dilution plating. C) Optical density of cultures. Untreated cells (Blue), FA treated cells (Red). Error shading represents the 95% confidence interval.
